# Preventing maternal deaths in Somalia requires stronger primary health care and functional emergency referral systems

**DOI:** 10.1016/j.pmedr.2026.103634

**Published:** 2026-09-12

**Authors:** Zakaria Abdi Abdullahi, Abdisalan Abdikarin Mohamed, Abdirahman Mohamed Adan, Ahmed Abdinasir Abdulle, Safia Shukri Hilowle

**Affiliations:** a Faculty of Health Sciences and Tropical Medicine, Somali National University, Mogadishu, Somalia; b Center for Health Research and Innovation, Somali National University, Mogadishu, Somalia; c Department of Waste Management, Ministry of Health, Federal Government of Somalia, Mogadishu, Somalia

## Abstract

Maternal mortality remains a critical public health crisis in Somalia, driven by structural vulnerabilities, low utilization of essential maternity care, and fragmented health service delivery. The vast majority of maternal deaths result from preventable obstetric complications such as hemorrhage, hypertensive disorders, sepsis, and obstructed labor. Current national surveys demonstrate severe gaps across the continuum of maternal care, particularly among rural, pastoralist, and internally displaced populations. Reducing preventable maternal deaths requires moving away from isolated emergency initiatives toward comprehensive health system strengthening. Priorities include scaling up primary health care facilities, expanding community midwifery networks, building organized emergency referral pathways with reliable communication and transport, and establishing maternal death surveillance systems. Connecting community-based health infrastructure directly to functional emergency obstetric facilities is essential to guarantee timely, life-saving care for pregnant women across Somalia.

Maternal mortality in Somalia remains among the highest globally despite ongoing national and international reconstruction efforts. Recent national epidemiological analyses reveal profound deficits across the maternity continuum of care. Only 9.7% of pregnant women receive at least four antenatal care visits, merely 2.8% complete adequate antenatal visits alongside skilled birth attendance, and a striking 78.1% of women receive no essential maternity services at all (Mohamoud et al., 2026). Furthermore, rates of facility-based deliveries remain exceptionally low, while unassisted home births persist as the default norm, particularly among geographically isolated and socioeconomically vulnerable communities (Mohamed et al., 2025; Osman et al., 2026).

Most maternal deaths in Somalia stem from direct, treatable obstetric complications, including postpartum hemorrhage, pre-eclampsia/eclampsia, puerperal sepsis, and obstructed labor (Mohamoud et al., 2026; Egal et al., 2023). These conditions can be managed effectively when women have access to timely, high-quality obstetric interventions. However, curbing maternal mortality requires more than expanding the sheer number of healthcare providers; it demands an integrated, functional health system linking communities, primary health care facilities, and referral centers capable of providing comprehensive emergency obstetric care (Mohamoud et al., 2026; Ahmed et al., 2024).

Weak primary health care infrastructure acts as a primary bottleneck by failing to facilitate routine antenatal visits, birth preparedness planning, and early identification of danger signs (Mohamoud et al., 2026). Evidence from Somalia and Somaliland indicates that women who participate in routine antenatal care are significantly more likely to deliver within health facilities (Osman et al., 2026; Egal et al., 2023). Conversely, extreme geographic distances, transport costs, and lack of health education severely hinder timely utilization of skilled maternity care (Osman et al., 2026; Egal et al., 2023; Ahmed et al., 2024). Inadequate birth preparedness further compounds vulnerability by delaying household-level decision-making when an emergency occurs (Ahmed et al., 2024).

Referral delays represent another major structural barrier to maternal survival (Ahmed et al., 2024; Yusuf, 2026). Transport deficiencies, weak communication channels between primary clinics and hospitals, poor inter-facility coordination, and inadequate clinical readiness contribute directly to preventable maternal fatalities (Mohamoud et al., 2026; Ahmed et al., 2024; Yusuf, 2026). In remote and pastoralist communities, women often endure prolonged delays reaching referral centers due to damaged road networks, lack of specialized ambulances, and fragmented referral mechanisms (Ahmed et al., 2024; Yusuf, 2026). Studies indicate that facility-based maternal deaths frequently occur following self-referral or late transfers, reflecting structural disorganization in care continuity (Ahmed et al., 2024).

These healthcare system challenges are further intensified by ongoing armed conflict, structural poverty, climate-related shocks, and massive population displacement. Pregnant women residing in internally displaced person settlements encounter severe barriers, including financial hardship, inadequate sanitation, heightened insecurity, and critical shortages of qualified midwives and basic medical supplies (Halane et al., 2026). Under such precarious conditions, delays in seeking and receiving medical attention are common, leading to poor maternal and perinatal outcomes (Halane et al., 2026).

Achieving meaningful reductions in maternal mortality in Somalia necessitates a transition from fragmented, project-based interventions toward systemic health-system strengthening. Essential strategy recommendations are summarized in Table 1.

**Table 1:** Key health system gaps and priority interventions for reducing maternal mortality in Somalia.

Health system domain	Identified barriers & gaps	Priority strategic interventions
Primary Health Care	Low antenatal care coverage (<10%); poor danger-sign awareness; high rates of unassisted home births.	Expand primary facilities; integrate community-wide birth preparedness education; subsidize essential maternity services.
Human Resources	Shortage of trained midwives in rural, pastoralist, and displaced communities.	Deploy certified midwives and community health workers to remote areas; provide continuous emergency obstetric training.
Emergency Referral Pathways	Fragmented communication; lack of emergency ambulances; long travel delays; high out-of-pocket transport costs.	Establish formal inter-facility communication protocols; deploy emergency transport networks; fund community emergency transport funds.
Facility Readiness	Shortages of blood banks, essential medicines (oxytocin, magnesium sulfate), and emergency surgical capacity.	Ensure continuous supply chain for basic and comprehensive emergency obstetric care; strengthen hospital infrastructure.
System Accountability	Absence of routine maternal mortality tracking and audit mechanisms.	Institutionalize Maternal Death Surveillance and Response (MDSR) to audit preventable deaths and drive quality improvements.

First, primary health care must be reinforced through active community engagement. Expanding access to routine antenatal care, promoting maternal danger-sign awareness, and encouraging birth preparedness are vital to increasing demand for skilled birth attendance (Mohamoud et al., 2026; Osman et al., 2026).

Second, the midwifery and community health worker workforce must be scaled up, particularly in rural, pastoralist, and displacement settings. Well-trained midwives stationed at primary health centers can identify pregnancy risks early, provide basic emergency care, and initiate timely transfers (Mohamed et al., 2025; Yusuf, 2026).

Third, establishing organized emergency referral systems is paramount. Sustainable investments are required to establish functional communication networks between primary care clinics and secondary referral hospitals, deploy equipped ambulances, and eliminate user-side transport costs during obstetric emergencies (Ahmed et al., 2024; Yusuf, 2026).

Fourth, emergency obstetric readiness must be upgraded across referral hospitals. This includes maintaining continuous supplies of safe blood for transfusions, essential medications (such as oxytocin and magnesium sulfate), surgical capacity, and skilled clinical teams (Mohamoud et al., 2026; Egal et al., 2023; Yusuf, 2026).

Finally, institutionalizing Maternal Death Surveillance and Response (MDSR) mechanisms across all administrative regions will provide actionable data to identify system bottlenecks, foster clinical accountability, and guide continuous quality improvement (Mohamoud et al., 2026; Egal et al., 2023).

In conclusion, preventing maternal deaths in Somalia requires a resilient primary health care foundation seamlessly connected to functional emergency referral networks and emergency obstetric care centers. Prioritizing these interconnected pillars must remain at the center of Somalia's national health policy agenda.

## CRediT authorship contribution statement

**Zakaria Abdi Abdullahi:** Conceptualization.

## Ethical approval

This commentary is based entirely on published literature and did not involve human participants or primary individual data collection. Ethical approval was not required.

## Funding

This work received no specific grant or financial support from any funding agency in the public, commercial, or not-for-profit sectors.

## Declaration of competing interest

The authors declare that they have no known competing financial interests or personal relationships that could have appeared to influence the work reported in this paper.

## Data Availability

No data was used for the research described in the article.
